# Capacitation induces changes in metabolic pathways supporting motility of epididymal and ejaculated sperm

**DOI:** 10.3389/fcell.2023.1160154

**Published:** 2023-06-27

**Authors:** Melanie Balbach, Lubna Ghanem, Sara Violante, Aye Kyaw, Ana Romarowski, Justin R. Cross, Pablo E. Visconti, Lonny R. Levin, Jochen Buck

**Affiliations:** ^1^ Department of Pharmacology, Weill Cornell Medicine, New York, NY, United States; ^2^ Donald B. and Catherine C. Marron Cancer Metabolism Center, Memorial Sloan Kettering Cancer Center, New York, NY, United States; ^3^ Department of Veterinary and Animal Science, Integrated Sciences Building, University of Massachusetts, Amherst, MA, United States

**Keywords:** capacitation, sperm maturation, hyperactivation, metabolic profiling, extracellular flux analysis

## Abstract

Mammalian sperm require sufficient energy to support motility and capacitation for successful fertilization. Previous studies cataloging the changes to metabolism in sperm explored ejaculated human sperm or dormant mouse sperm surgically extracted from the cauda epididymis. Due to the differences in methods of collection, it remains unclear whether any observed differences between mouse and human sperm represent species differences or reflect the distinct maturation states of the sperm under study. Here we compare the metabolic changes during capacitation of epididymal *versus* ejaculated mouse sperm and relate these changes to ejaculated human sperm. Using extracellular flux analysis and targeted metabolic profiling, we show that capacitation-induced changes lead to increased flux through both glycolysis and oxidative phosphorylation in mouse and human sperm. Ejaculation leads to greater flexibility in the ability to use different carbon sources. While epididymal sperm are dependent upon glucose, ejaculated mouse and human sperm gain the ability to also leverage non-glycolytic energy sources such as pyruvate and citrate.

## Introduction

Mammalian sperm leaving the male reproductive tract upon ejaculation are morphologically mature but functionally immature and cannot fertilize the oocyte. During ejaculation, sperm mix with seminal and prostatic fluids containing elevated levels of bicarbonate. Bicarbonate stimulates Soluble Adenylyl Cyclase (sAC; ADCY10), which activates sperm motility and initiates the maturation processes required for successful fertilization (i.e., capacitation) (Chang, 1951; Austin and Bishop, 1958). The early molecular changes during capacitation, including increased levels of intracellular cAMP and calcium (Gervasi and Visconti, 2016; Balbach et al., 2018), are followed by functional changes. Freshly ejaculated sperm exhibit a low-amplitude and symmetrical flagellar beat, resulting in a progressive movement called activated motility (Publicover et al., 2007; Dey et al., 2019). During capacitation, sperm modify their motility pattern into an asymmetric flagellar beat, representing a swimming mode termed hyperactivation (Yanagimachi, 1970; Ishijima et al., 2002). Hyperactivation is a fast whip-like movement of the flagellum with an irregular trajectory, which generates the propulsive forces that are needed for sperm to reach the oocyte and overcome its vestments; i.e., the cumulus oophorous and the zona pellucida (Ho and Suarez, 2001; Suarez, 2008). Hyperactivation may also facilitate penetration of the mucus in the oviductal lumen and the release of sperm from the oviductal storage reservoir.

Sperm motility is directly dependent upon the availability of chemical energy obtained through ATP hydrolysis and accounts for the majority of ATP consumed (Bohnensack and Halangk, 1986). Thus, mammalian sperm are dependent on sufficient energy production to maintain flagellar movement within the female genital tract and to complete their journey to the oocyte (reviewed in (Visconti, 2012; du Plessis et al., 2015)). Mammalian sperm possess the metabolic machinery to generate ATP and other compounds supplying chemical energy via both glycolysis and the mitochondrial Krebs cycle and oxidative phosphorylation (oxphos). Metabolic studies demonstrated that externally supplied glycolytic substrates are sufficient to support *in vitro* capacitation, acrosomal exocytosis, progressive motility, and hyperactivation of sperm from both men and mice (Peterson and Freund, 1970; Hoppe, 1976; Fraser and Quinn, 1981; Rogers and Perreault, 1990; Williams and Ford, 2001; Mukai and Okuno, 2004; Goodson et al., 2012). However, it remains unclear how each species specifically utilizes its metabolic machinery to generate ATP for each of these energy-demanding processes. For example, while mouse sperm ceased moving after 30–40 min in media without an exogenous energy source (Navarrete et al., 2019), human sperm remained motile for at least 24 h in the absence of exogenous nutrients (Suter et al., 1979; Ford and Harrison, 1981; Navarrete et al., 2019; Carrageta et al., 2020; Marín-Briggiler et al., 2021), suggesting they possess stored energy reserves. Comparative studies also identified a difference between mouse and human sperm’s ability to utilize exogenous pyruvate. While mouse sperm seemed surprisingly unaffected by pyruvate supplementation (Balbach et al., 2020a), addition of pyruvate to glucose-containing media enhances progressive motility and hyperactivation of human sperm (Hereng et al., 2011).

Metabolic discrepancies between mouse and human sperm may reflect species-specific metabolic requirements or regulation. Alternatively, these differences may derive from the experimental conditions utilized. Previous experiments studied human sperm purified from ejaculated semen. The fluid portion of semen, seminal plasma, contains high amounts of fructose and citrate, in addition to glucose, lactate, and free amino acids, and the bicarbonate which initiates the molecular changes of capacitation (Owen and Katz, 2005). Human sperm metabolism was studied in sperm separated from seminal plasma prior to experiments; however, these studies assessed metabolism after human sperm were exposed to these metabolites, and after the bicarbonate in seminal plasma initiated capacitation. In contrast, mouse sperm metabolic experiments have been performed on sperm surgically extracted from the cauda epididymis, which are stored in a dormant, inactivated state, exposed only to the purification media, right until the start of the experiment. These different metabolic and activation states complicate interpretations of experiments comparing dormant epididymal mouse sperm with ejaculated human sperm. Ejaculated mouse sperm can be isolated from the female genital tract following copulation (YANAGIMACHI and CHANG, 1963; Whittingham, 1968; King et al., 1994). Like ejaculated human sperm, mouse sperm recovered from the female genital tract were exposed to the metabolites and activating bicarbonate levels in semen. Ejaculated mouse sperm isolated from the female tract proved informative in a recent study of the sperm Ca^2+^ channel CatSper. Activation of CatSper had been thought to differ between mouse and human; however, by comparing epididymal *versus* ejaculated mouse with human sperm, CatSper activation was shown to be conserved between mouse and human sperm, and was dependent on the sperm maturation state (Ferreira et al., 2021a). These findings motivate a detailed analysis of the metabolic pathways utilized during capacitation in human sperm compared to mouse sperm before and after ejaculation.

We recently developed a minimally invasive technique using an extracellular flux analyzer to observe real-time changes in sperm metabolism during capacitation (Balbach et al., 2020b). Focusing exclusively on recently dormant epididymal mouse sperm, we showed that both glycolysis and oxphos increase during capacitation (Balbach et al., 2020a). The increase in both metabolic pathways was dependent on glycolytic substrates, and the increase in oxphos was dependent on glycolysis (Balbach et al., 2020a), providing evidence for a direct link between oxphos and glycolysis in sperm. Here we extend these results by using extracellular flux analysis and targeted metabolic profiling to compare the metabolic requirements for capacitation between ejaculated human sperm, epidydimal mouse sperm and ejaculated mouse sperm recovered from the female genital tract. We find that among the metabolic changes during ejaculation, sperm gain greater flexibility in utilizing diverse carbon sources.

## Results

### Epididymal mouse sperm, uterine mouse sperm and ejaculated human sperm rely on both glycolysis and oxphos to generate chemical energy

**FIGURE 1:**
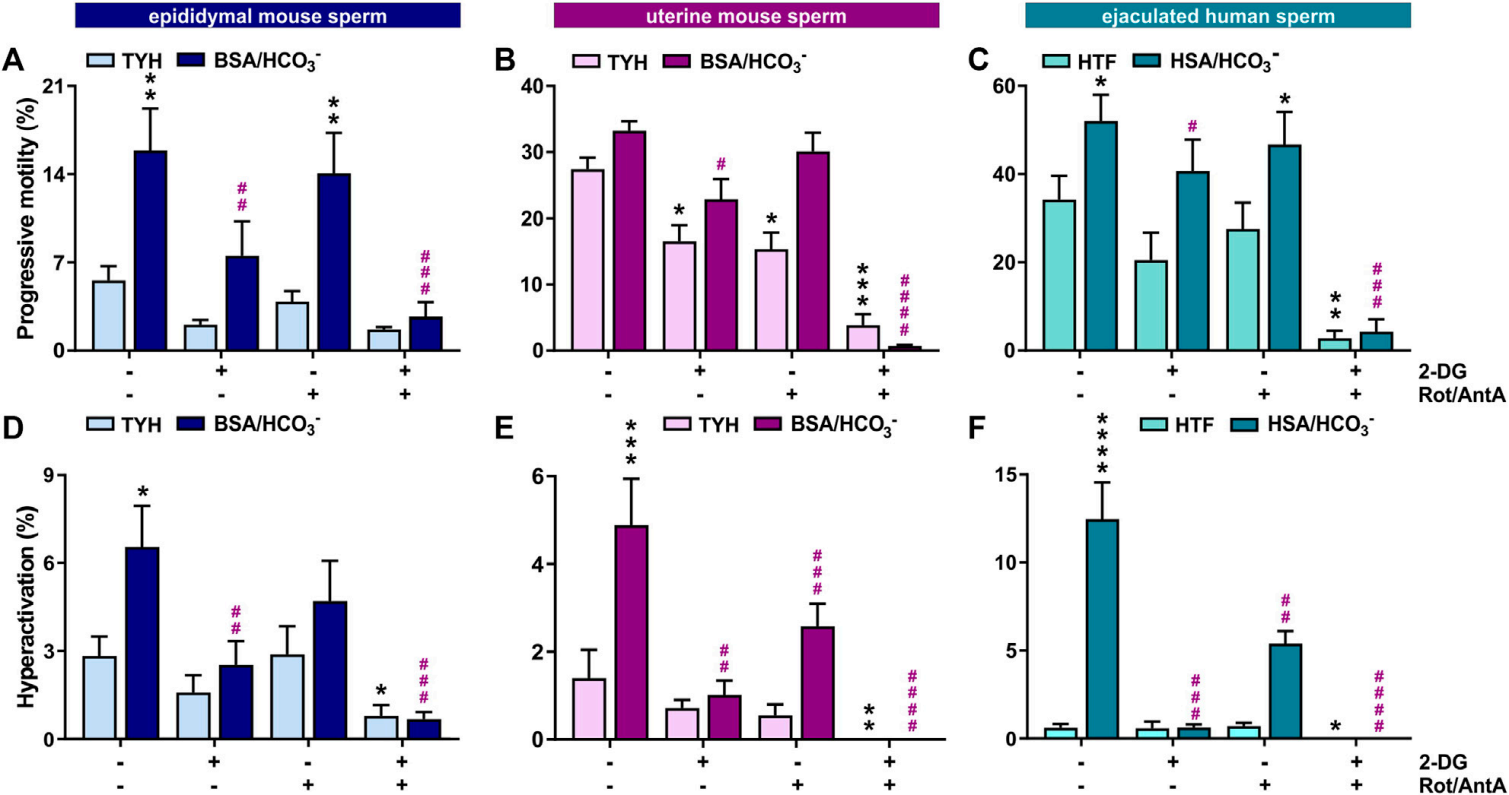
In mouse and human sperm, hyperactivated motility is dependent on glycolysis and oxphos **(A–C)** Percentage of progressively motile **(A)** epididymal mouse sperm, **(B)** uterine mouse sperm, or **(C)** ejaculated human sperm incubated for 30 min (mouse) or 60 min (human) in non-capacitating (mouse sperm: TYH, human sperm: HTF) or capacitating media (mouse sperm: TYH + BSA + HCO_3_^−^, human sperm: HTF + HSA + HCO_3_^−^) with glucose (mouse) or glucose/pyruvate (human) in the presence or absence of 2-DG (mouse: 56 mM, human: 28 mM) and/or Rot/AntA (0.5 μM); mean +SEM (n ≥ 7). **(D–F)** Percentage of hyperactivated **(D)** epididymal mouse sperm, **(E)** uterine mouse sperm, or **(F)** ejaculated human sperm incubated for 30 min (mouse) or 60 min (human) in non-capacitating or capacitating media in the presence or absence of 2-DG or AntA/Rot; mean +SEM (n ≥ 7). Differences between conditions were analyzed using one-way ANOVA compared to the DMSO-treated non-capacitated (black asterisk) or capacitated (purple pound sign) control,*p < 0.05, **p < 0.01, ***p < 0.001, ****p < 0.0001.

Motility is the major consumer of ATP in activated sperm (Bohnensack and Halangk, 1986). We used computer-assisted sperm analysis (CASA) to track, quantify and compare motility of *Mus musculus* mouse sperm isolated from the cauda epididymis (epidydimal mouse sperm), mouse sperm ejaculated into the female uterus (uterine mouse sperm), and ejaculated human sperm incubated in non-capacitating (mouse: TYH, human: HTF) and conditions which induce molecular hallmarks of capacitation (i.e., elevated cAMP, increased progressive and hyperactivated motility) (mouse: TYH + BSA + HCO_3_^−^, human: HTF + HSA + HCO_3_^−^). Mammalian sperm possess the molecular machinery to generate ATP via glycolysis and oxidative phosphorylation (oxphos). By inhibiting glycolysis with 2-deoxyglucose (2-DG) or blocking the electron transport chain with rotenone (Rot) and antimycin A (AntA), we were able to compare the relative contributions of glycolysis and oxphos to progressive and hyperactive motility. In all sperm samples, the percentage of hyperactivated sperm increased after exposure to media which induces capacitation hallmarks containing glucose (mouse sperm) and glucose/pyruvate (human sperm) as energy source. The percentage of progressively motile sperm increased in epididymal mouse and ejaculated human sperm in capacitating media. The level of progressively motile sperm in uterine mouse sperm was elevated in non-capacitating conditions (Figures 1A, B); this difference may represent a physiological change induced by motility-enhancing factors in the seminal plasma or female genital tract, and/or it may be due to selection of motile sperm during ejaculation. Human sperm exhibited the highest percentage of progressively motile sperm (Figure 1C). Whether this difference reflects a true distinction between the species is unclear because the progressive motility of C57Bl/6 *M. musculus* sperm is low relative to other mouse species (Odet et al., 2008; Park et al., 2012; Fan et al., 2015; Tourmente et al., 2015) and motile human sperm are enriched during purification from semen. Inhibiting glycolysis greatly diminished the percentage of progressively motile sperm in capacitating conditions in all three sperm samples, consistent with previous studies of epididymal mouse sperm (Mukai and Okuno, 2004; Goodson et al., 2012). In all three samples, in capacitating conditions motility was unaffected when oxphos was blocked, suggesting that oxphos is not essential for generating the ATP which supports motility; however, when both glycolysis and oxphos were inhibited, progressive motility was reduced to nearly zero. This indicates that the ATP generated via oxphos can support at least some progressive motility, particularly when glycolysis is inactive.

Hyperactivated motility is the asymmetric, more powerful flagellar beating that increases energy demand. In all three sperm samples, the capacitation-induced stimulation of hyperactivated motility was entirely dependent upon glycolysis and partially dependent upon oxphos (Figures 1D–F), and as expected, hyperactivated motility was completely absent when both pathways were blocked. Thus, both glycolysis and oxphos can contribute ATP to support motility, and there appears to be synergy between the two pathways in sperm. Particularly during hyperactivated motility with its high energy demand, chemical energy is supplied by the combination of glycolysis and oxphos.

### Glycolysis and oxidative phosphorylation increase during capacitation of epididymal mouse sperm, uterine mouse sperm and ejaculated human sperm

We used an extracellular flux analyzer to compare how glycolysis and oxphos change during capacitation in epididymal and uterine mouse sperm as well as ejaculated human sperm. The Seahorse XFe96 extracellular flux analyzer detects the extracellular acidification rate (ECAR), i.e., H^+^ extruded into the assay media coupled to export of glycolysis-generated lactate via proton-driven monocarboxylate transporters (Brauchi et al., 2005)^,^ (Lee et al., 2014), to reflect changes in glycolysis. At the same time, it measures the oxygen consumption rate (OCR) to quantitate the uptake of O_2_ used by oxphos (Balbach et al., 2020b). Because the system’s sensitivity to pH changes prevents inducing capacitation via the physiological stimulant bicarbonate, we capacitated sperm by adding the cell-permeable cAMP analog db-cAMP together with the broad-specificity phosphodiesterase inhibitor IBMX. This combination initiates capacitation-induced changes, specifically the progesterone-induced acrosome reaction, as effectively as addition of HCO_3_^−^ to both mouse (Balbach et al., 2020a) and human sperm (Supplementary Figure S1).

**FIGURE 2:**
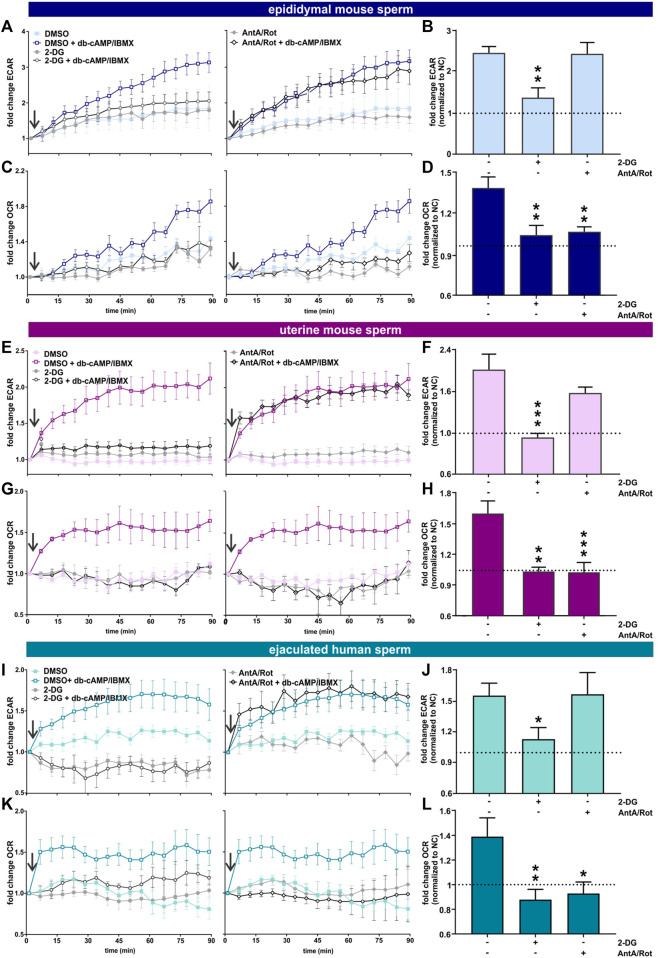
Capacitation of epididymal and ejaculated mouse and human sperm is accompanied by increased glycolysis and oxphos **(A, E, I)** Normalized ECAR of **(A)** epididymal mouse sperm, **(E)** uterine mouse sperm, or **(I)** ejaculated human sperm in non-capacitating or capacitating (db-cAMP + IBMX) media in the absence (DMSO) or presence of (left) 2-DG or (right) AntA/Rot; mean ± SEM (n ≥ 6). **(B,F, J)** Change in ECAR of **(B)** epididymal mouse sperm, **(F)** uterine mouse sperm, or **(J)** ejaculated human sperm in capacitating media relative to control in non-capacitating media in the absence or presence of 2-DG or AntA/Rot. The average of the last three data points was respectively used to calculate the fold change. **(C,G, K)** Normalized OCR of **(C)** epididymal mouse sperm, **(G)** uterine mouse sperm, or **(K)** ejaculated human sperm in non-capacitating or capacitating (db-cAMP + IBMX) media in the absence (DMSO) or presence of (left) 2-DG or (right) Rot/AntA; mean ± SEM (n ≥ 6) **(D, H, L)** Change in OCR of **(D)** epididymal mouse sperm, **(H)** uterine mouse sperm, or **(L)** ejaculated human sperm in capacitating media relative to control in non-capacitating media in the absence or presence of 2-DG or AntA/Rot; mean +SEM (n ≥ 6). The average of the last three data points was respectively used to calculate the fold change. Differences between conditions were analyzed using one-way ANOVA compared to the DMSO-treated capacitated control **(B, D, F, H, J, L)**,*p < 0.05, **p < 0.01, ***p < 0.001. The arrow indicates addition of 5 mM db-cAMP/500 µM IBMX.

As shown previously (Tourmente et al., 2015; Balbach et al., 2020a), in exogenously-supplied glucose in non-capacitating conditions, epididymal mouse sperm exhibited a gradual increase in ECAR and OCR over time, possibly reflecting activation of their metabolic machinery. For uterine mouse sperm and ejaculated human sperm in non-capacitating conditions ECAR and OCR remained mostly stable over time. In all sperm samples, ECAR and OCR increased when capacitation was mimicked by exposure to cAMP (Figure 2), and the kinetics of the cAMP-induced changes differed depending upon the activation state of the sperm sample. Epididymal mouse sperm, which were never previously exposed to capacitating conditions, exhibited the strongest cAMP-induced stimulation in ECAR and OCR with a steady increase over time that did not saturate for the duration of the measurement (Figures 2A, C). In contrast, ECAR increased with a faster kinetic and saturated 60 min post injection of db-cAMP/IBMX in both uterine mouse sperm and ejaculated human sperm (Figures 2E, I). For OCR, in ejaculated sperm, we detected a strong initial increase post exposure to cAMP that saturated within 10 min (ejaculated human sperm) and 20 min (uterine mouse sperm) (Figures 2C, G, K). The faster ECAR and OCR kinetics suggest uterine mouse sperm and ejaculated human sperm start at a different metabolic state than epididymal mouse sperm when placed into the extracellular flux analyzer. Thus, while uterine mouse and ejaculated human sperm already had their metabolic pathways stimulated during ejaculation, epididymal mouse sperm start from a lower baseline of metabolic activity (Supplementary Table S1); hence, their activation kinetics is slower. Epididymal mouse sperm experience the largest capacitation-induced response presumably because these sperm were, until recently, stored in a dormant state with little metabolic activity.

In all sperm samples, the cAMP-induced changes in ECAR were prevented when glycolysis was blocked by 2-DG and unaffected by Rot/AntA (Figures 2A, B, E, F, I, J). Thus, the capacitation-induced changes in ECAR are driven by glycolysis with little contribution from oxphos. As expected, inclusion of Rot/AntA blocked changes in OCR in epididymal and uterine mouse sperm and ejaculated human sperm (Figures 2C, D, G, H, K, L), confirming that capacitation-induced increases in OCR are due to oxphos. In all three sperm samples, in the presence of a glycolytic energy source, changes in OCR were blocked by 2-DG, suggesting, as reported previously for epididymal mouse sperm (Balbach et al., 2020a), that the capacitation-induced increase in oxphos is fueled by glycolytic activity. This dependence establishes a functional link between these pathways in both human and mouse sperm before and after ejaculation. In summary, starting with ejaculation and continuing through capacitation, human and mouse sperm stimulate glycolytic activity leading to a concomitant increase in oxygen consumption. Depending upon the amount of uncoupling present in sperm mitochondria (Ferramosca et al., 2013; Carrageta et al., 2023), this increased oxygen consumption will translate into increased oxphos to support the greater energy demands of capacitating sperm.

### Metabolite profiling confirms increased rates of glycolysis and oxphos during capacitation

**FIGURE 3:**
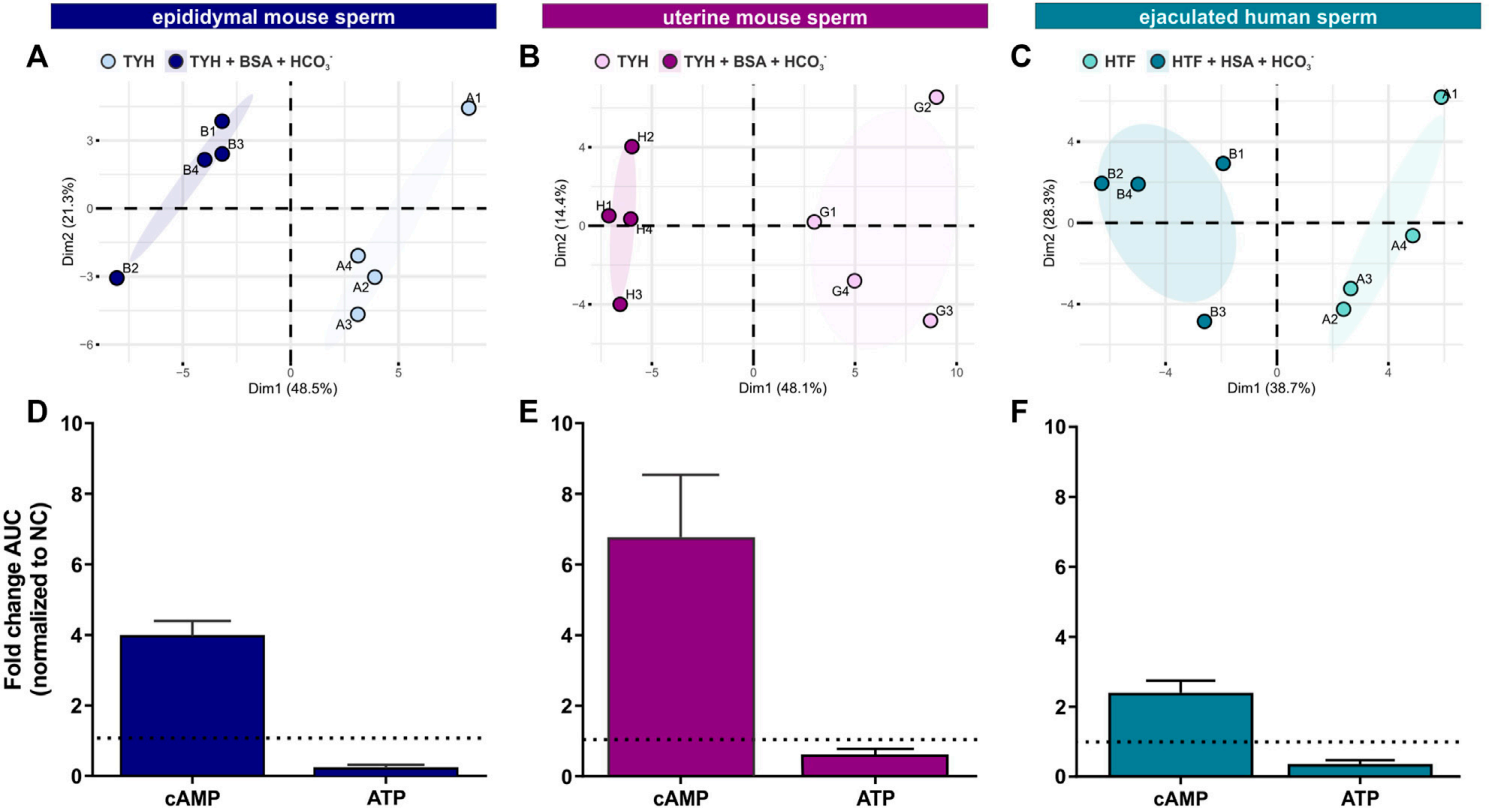
Sperm intracellular metabolite levels during capacitation are a product of production and consumption **(A–C)** Principal component analysis (PCA) plots of metabolites isolated from **(A)** epididymal mouse sperm (PC1 score p = ***, PC2 score p = n.s.), **(B)** uterine mouse sperm (PC1 score p = **, PC2 score p = n.s.), or **(C)** ejaculated human sperm (PC1 score p = **, PC2 score p = n.s.) incubated for 90 min in non-capacitating or capacitating media. To highlight the different cluster, sperm incubated in non-capacitating and capacitating conditions are surrounded by eclipses; representative plots are shown **(D–F)** Nucleotides detected in **(D)** epididymal mouse sperm, **(E)** uterine mouse sperm, or **(F)** ejaculated human sperm incubated for 90 min in capacitating conditions in glucose, fold change of integrated area under the peak (AUC) normalized to the non-capacitated control; mean +SEM (n = 3). Differences between conditions **(A–C)** were analyzed using two-tailed, paired *t*-test, **p < 0.01, ***p < 0.001, n. s = not significant.

To complement the real-time measurements via extracellular flux analysis, we utilized mass spectrometry and metabolic profiling to understand how individual endogenous metabolites change upon exposure to capacitating conditions. Unsupervised hierarchical clustering of metabolites extracted with methanol, analyzed via LC-MS/MS and confirmed using biochemical standards (all metabolites detected are listed in Tab. 1–6) revealed distinct patterns for epididymal and uterine mouse sperm and ejaculated human sperm incubated in non-capacitating and capacitating conditions for 90 min in the presence of glucose (Figures 3A–C).

**FIGURE 4:**
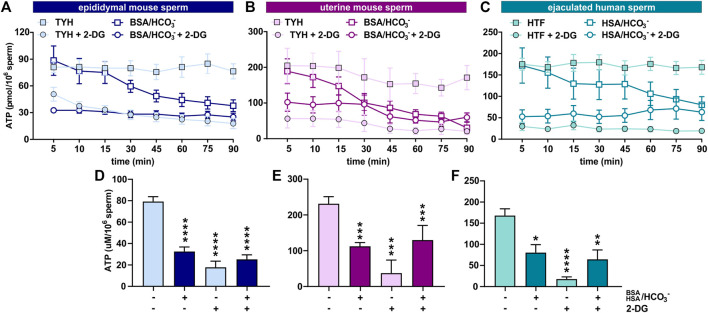
Sperm intracellular ATP levels decrease during capacitation **(A–C)** Intracellular ATP levels in **(A)** epididymal mouse sperm, **(B)** uterine mouse sperm, or **(C)** ejaculated human sperm at different time points during incubation in non-capacitating or capacitating media in the presence or absence of 2-DG; mean ± SEM (n ≥ 5) **(D–F)** Intracellular ATP levels in **(D)** epididymal mouse sperm, **(E)** uterine mouse sperm, or **(F)** ejaculated human sperm incubated in non-capacitating or capacitating media for 90 min in the presence or absence of 2-DG; mean ± SEM (n ≥ 5). Differences between conditions **(D–F)** were analyzed using one-way ANOVA compared to the non-capacitated control in DMSO, *p < 0.05, **p < 0.01, ***p < 0.001, ****p < 0.0001.

**FIGURE 5:**
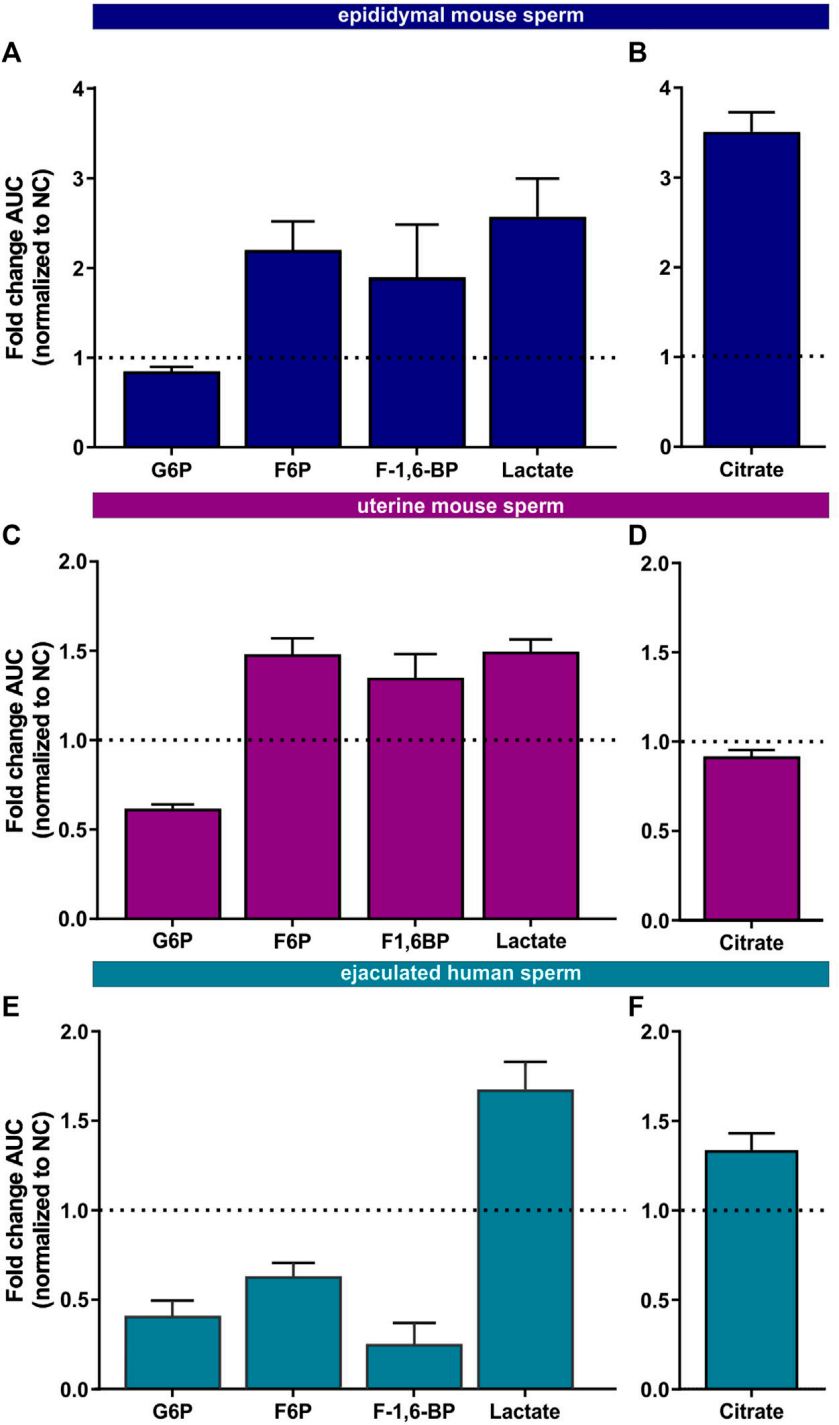
Metabolic profiling of mammalian sperm in non-capacitated and capacitating conditions **(A,C, E)** Glycolytic metabolites detected in **(A)** epididymal mouse sperm, **(C)** uterine mouse sperm, or **(E)** ejaculated human sperm incubated for 90 min in capacitating conditions in glucose, fold change AUC normalized to the non-capacitated control; mean +SEM (n = 3) **(B, D, F)** Citrate detected in **(B)** epididymal mouse sperm, **(D)** uterine mouse sperm, or **(F)** ejaculated human sperm incubated for 90 min in capacitating conditions, fold change AUC normalized to the non-capacitated control; mean +SEM (n = 3). G6P = glycerol-6-phosphate, F6P = fructose-6-phosphate, F-1,6-BP = fructose 1,6-bisphosphate.

Initially, we explored the levels of two intracellular metabolites predicted to change in capacitating sperm incubated in glucose and glucose/pyruvate. As expected, levels of intracellular cAMP, the earliest signaling pathway activated in capacitation (Wennemuth et al., 2003; Buffone et al., 2014; Balbach et al., 2018), were elevated in all sperm samples after exposure to capacitating conditions (Figures 3D–F, Supplementary Figures S2A, D, G). We previously showed that capacitating conditions stimulate ATP generation due to increased glucose uptake (Hidalgo et al., 2020) and utilization (Balbach et al., 2020a). However, ATP levels were lower in sperm incubated in capacitating conditions compared to the levels in sperm maintained in non-capacitating conditions (Figures 3D–F, Supplementary Figures S2A, D, G). We independently confirmed this change in ATP by directly measuring intracellular ATP over time. Consistent with metabolite profiling, ATP levels decreased in all sperm samples incubated in capacitating conditions, and as expected, ATP levels were dependent upon glycolytic activity; inhibiting glycolysis with 2-DG rapidly reduced ATP levels in both non-capacitating and capacitating conditions (Figures 4A–F). These data illustrate the important concept that metabolite levels measured by profiling, including cAMP and ATP, reflect a balance between production and consumption, and changes in metabolism during capacitation can result in increased or decreased metabolite levels. Thus, the reduced ATP levels in capacitating sperm, despite increased production, reveal that the rate of ATP consumption during capacitation exceeds the stimulated production resulting in a lower ATP equilibrium. These data confirm that this metabolic profiling methodology is sufficiently stringent and sensitive to distinguish non-capacitating from capacitating sperm. To independently confirm the capacitation-induced changes in the rates of glycolysis and oxphos predicted by extracellular flux analysis, we focused on individual metabolites involved in glycolysis and the TCA cycle. Levels of the glycolytic intermediate glucose-6-phosphate in both glucose (Figures 5A, C, E) and glucose/pyruvate (Supplementary Figures S2B, E, H) were reduced following exposure to capacitating conditions in human sperm and in mouse sperm before and after ejaculation. Similarly, and as previously reported in epididymal mouse sperm and in line with extracellular flux analysis (Calvert et al., 2019; Carrageta et al., 2020), all sperm samples incubated in capacitating conditions showed increased levels of lactate, the product of anaerobic glycolysis. Levels of other glycolytic intermediates also changed following capacitation, confirming that capacitation affects glycolytic rates, but there were potentially interesting species-specific differences. In human sperm, in both glucose and glucose/pyruvate fructose-6-phosphate and fructose-1,6-bisphosphate levels were decreased following capacitation, while these metabolites were elevated following capacitation in glucose in both epididymal and uterine mouse sperm. Interestingly, in glucose/pyruvate fructose-1,6-bisphosphate levels were decreased in epididymal and uterine mouse sperm. Investigating these potentially revealing species-specific differences and differences between glucose and glucose/pyruvate will require metabolic tracing studies monitoring flux of exogenously supplied glucose labeled with stable isotopes. Regarding lower glycolysis, capacitated epididymal and uterine mouse sperm had decreased levels of 2-phosphoglycerate and phosphoenolpyruvate (Supplementary Tables S1, S2, S4, S5). These metabolites were below the limit of detection in human sperm (Supplementary Tables S3, S6).

We also found changes in Krebs cycle metabolites in all three sperm samples in both glucose and glucose/pyruvate (Figures 5B, D, F, Supplementary Figures S2C, F, I) confirming the real-time observation that oxphos increases during capacitation. Human sperm accumulate citrate during capacitation. For mouse sperm, we identified a difference in Krebs cycle metabolism based upon activation state when incubated in glucose. While epididymal mouse sperm accumulate citrate during capacitation, citrate levels decreased following capacitation of uterine mouse sperm. However, when pyruvate was added to glucose, citrate accumulated in uterine sperm. These data suggest that the citrate equilibrium in uterine mouse sperm is altered when sperm additionally metabolize pyruvate.

### Post ejaculation, sperm can leverage exogenously supplied substrates which enter oxphos directly

**FIGURE 6:**
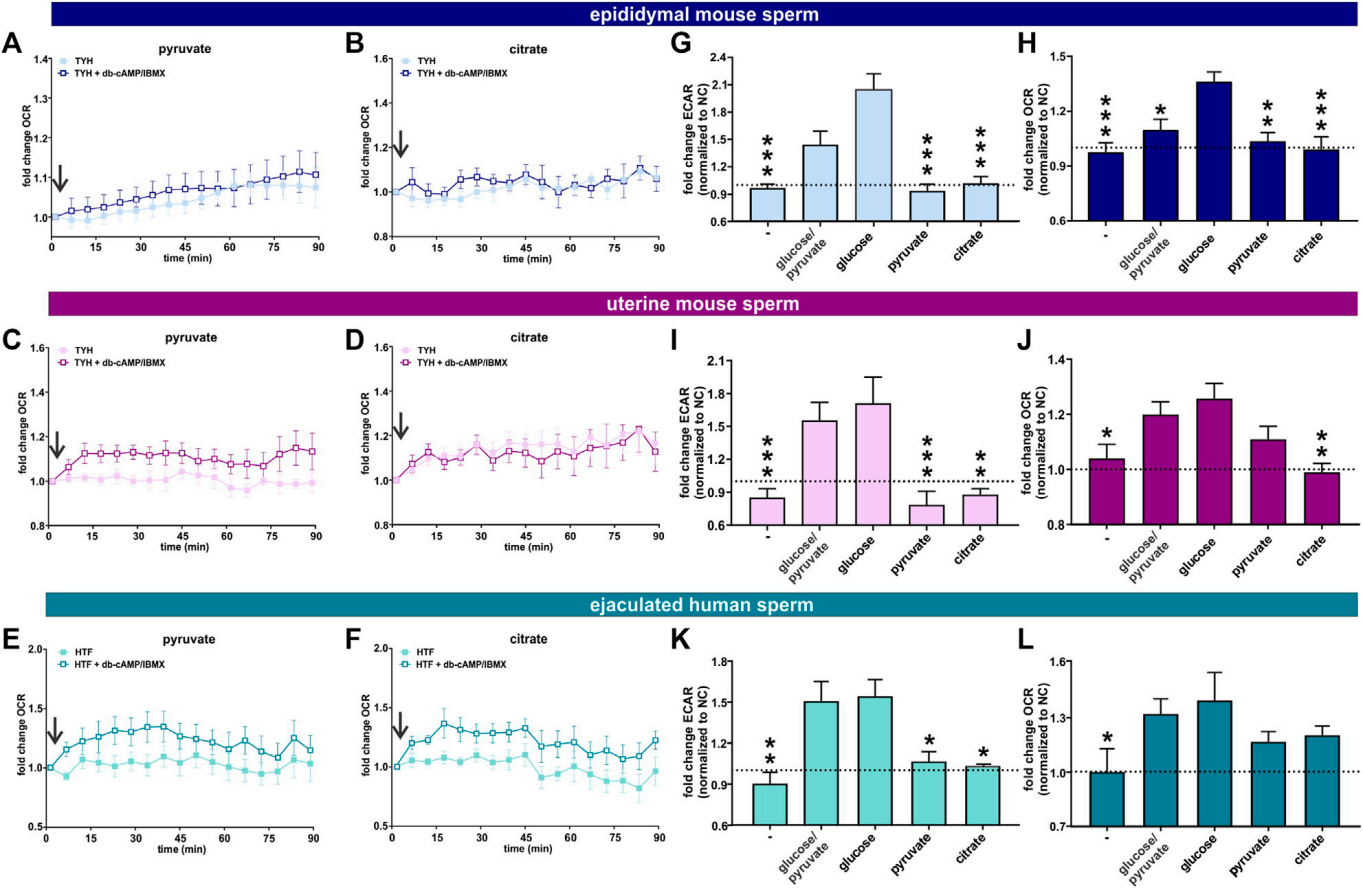
Post ejaculation, non-glycolytic substrates can support the capacitation-induced changes in metabolism **(A, B)** Normalized OCR of epididymal mouse sperm in non-capacitating or capacitating media with **(A)** pyruvate and **(B)** citrate; mean ± SEM (n ≥ 7) **(C, D)** Normalized OCR of uterine mouse sperm in non-capacitating or capacitating media with **(C)** pyruvate and **(D)** citrate; mean ± SEM (n ≥ 7) **(E, F)** Normalized OCR of ejaculated human sperm in non-capacitating or capacitating media with **(E)** pyruvate and **(F)** citrate; mean ± SEM (n ≥ 7). Arrow indicates addition of 5 mM db-cAMP/500 µM IBMX. **(G, I, K)** Change in ECAR of capacitated **(G)** epididymal mouse sperm, **(I)** uterine mouse sperm, or **(K)** ejaculated human sperm relative to non-capacitated control in the presence of no exogenous energy source (−), glucose/pyruvate, glucose, pyruvate, or citrate; mean +SEM (n ≥ 7) **(H, J, L)** Change in OCR of capacitated **(H)** epididymal mouse sperm **(J)** uterine mouse sperm, or **(L)** ejaculated human sperm relative to non-capacitated control in the presence of no exogenous energy source (−), glucose/pyruvate, glucose, pyruvate, or citrate; mean +SEM (n ≥ 7). The average of the last three data points was respectively used to calculate the fold change. Differences between conditions were analyzed using one-way ANOVA compared to sperm incubated in glucose **(G–L)**,*p < 0.05, **p < 0.01, ***p < 0.001. The arrow indicates addition of 5 mM db-cAMP/500 µM IBMX.

**FIGURE 7:**
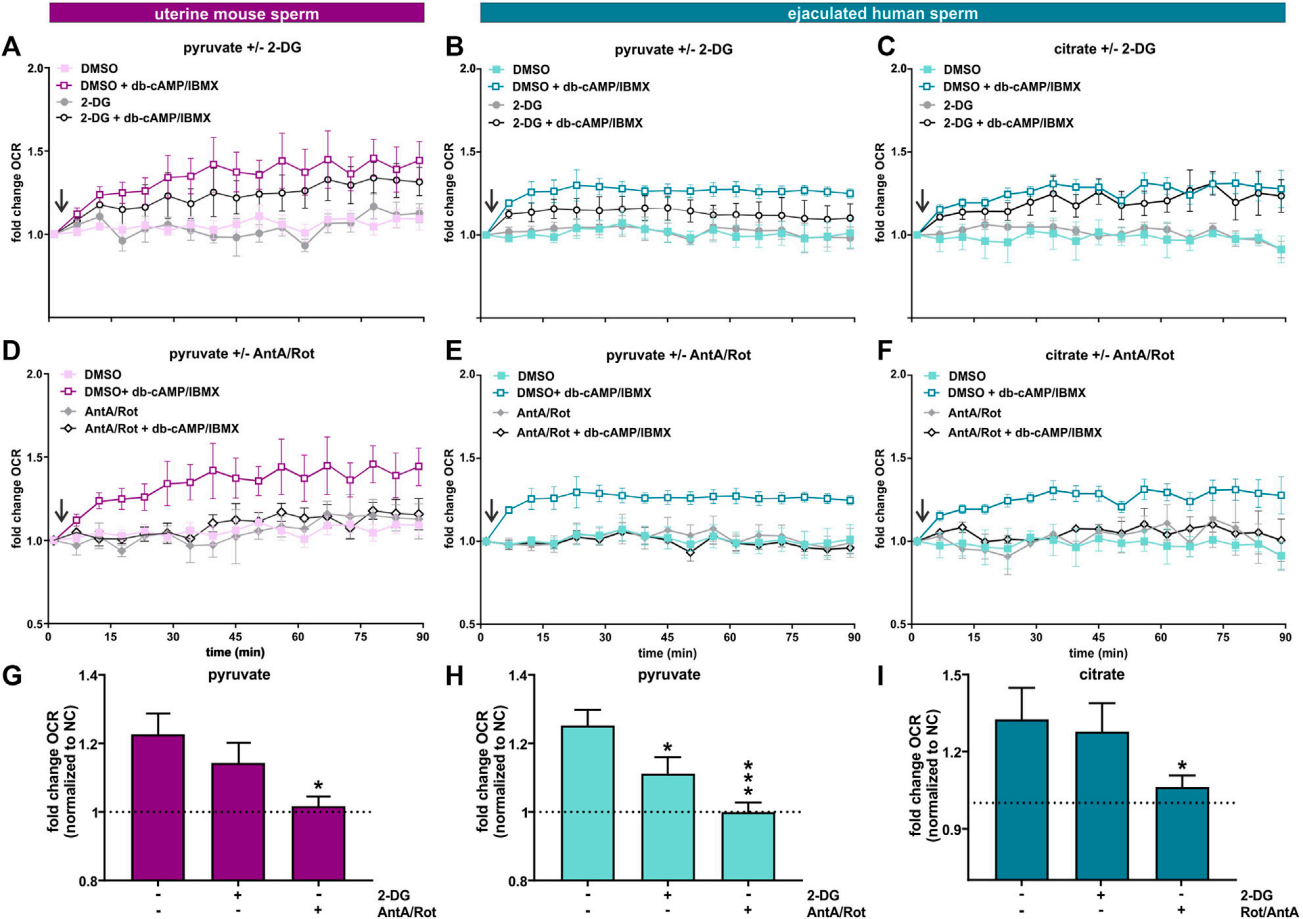
Capacitation induces an increase in oxphos independent from glycolysis **(A, D)** Normalized OCR of uterine mouse sperm in non-capacitating or capacitating media with pyruvate in the presence and absence of **(A)** 2-DG or **(D)** AntA/Rot; mean ± SEM (n ≥ 6). **(B, E)** Normalized OCR of ejaculated human sperm in non-capacitating or capacitating media with pyruvate in the presence and absence of **(B)** 2-DG or **(E)** AntA/Rot; mean ± SEM (n ≥ 6) **(C, F)** Normalized OCR of ejaculated human sperm in non-capacitating or capacitating media with citrate in the presence and absence of **(C)** 2-DG or **(F)** AntA/Rot; mean ± SEM (n ≥ 6). **(G)** Change in OCR of uterine mouse sperm in capacitating media containing pyruvate relative to control in non-capacitating media in the absence or presence of 2-DG or AntA/Rot **(H, I)** Change in OCR of ejaculated human sperm in capacitating media containing **(H)** pyruvate or **(I)** citrate relative to control in non-capacitating media in the absence or presence of 2-DG or AntA/Rot. The average of the last three data points was respectively used for normalization, mean +SEM (n ≥ 6). Differences between conditions were analyzed using one-way ANOVA compared to the DMSO-treated capacitated control **(G–I)**,*p < 0.05, ***p < 0.001. The arrow indicates addition of 5 mM db-cAMP/500 µM IBMX.

In our previous studies focusing on epididymal mouse sperm, we demonstrated that the capacitation-induced increase in oxphos required substrates generated intrinsically from glycolysis: Fuels which bypass glycolysis and enter the Krebs cycle directly were unable to support the capacitation-induced increase in OCR (Balbach et al., 2020a). Here, we explore whether uterine mouse sperm and ejaculated human sperm, which have already encountered glycolytic substrates during ejaculation (Mann, 1965; Purvis et al., 1986; Owen and Katz, 2005), maintain a similar dependence. As anticipated, epididymal and uterine mouse sperm as well as ejaculated human sperm required exogenous nutrients to support the increase in glycolysis and oxphos elicited by the addition of db-cAMP/IBMX (Figures 6G–L) and including glucose in the extracellular flux analyzer media resulted in the strongest increase in both glycolysis and oxphos in all three sperm samples (Figures 6G–L, Supplementary Figure S3). As we previously showed, exogenous addition of pyruvate, which cannot, by itself, support an increase in glycolysis (Figures 6G, I, K), did not increase OCR in epididymal mouse sperm; they are dependent upon pyruvate generated intrinsically from glycolysis to support the capacitation-induced change in oxphos (Balbach et al., 2020a) (Figures 6A,H). Surprisingly, in both post-ejaculated human and uterine mouse sperm, OCR was stimulated upon addition of db-cAMP/IBMX in media containing pyruvate only (Figures 6C, E, J, L). This increase in OCR was prevented by Rot/AntA confirming it represents oxphos activity, and it was also partly diminished by the glycolysis inhibitor 2-DG (Figures 7A, B, D, E, G, H), supporting the existence of a functional link between glycolysis and oxphos. Also as we described previously (Balbach et al., 2020a), adding pyruvate to glucose diminished both ECAR and OCR responses relative to glucose alone in epidydimal mouse sperm suggesting that pyruvate feeds back and partially inhibits glycolysis and oxphos. This effect was not seen in uterine mouse sperm and ejaculated human sperm; addition of pyruvate did not appreciably affect the capacitation-induced response (Figures 6I–K, L, Supplementary Figure S3). The ability of uterine mouse and ejaculated human sperm to increase OCR in the presence of exogenously supplied pyruvate reveals that the cAMP-dependent stimulation of oxphos does not simply reflect increased substrate availability (i.e., pyruvate) produced from the elevated glycolytic rate; there appears to be an independent, specific stimulation of oxphos activity in capacitating sperm post ejaculation.

The TCA cycle intermediate citrate presents a unique profile. Only human sperm were able to utilize citrate for the capacitation-induced increase in oxphos (Figures 6B, D, F, H , J, L). As expected, this increase in OCR was prevented by Rot/AntA and unaffected by 2-DG (Figures 7C, F, I). Despite being part of the Krebs cycle, citrate is not permeable to mitochondria, it first needs to be converted to malate. Human sperm appear to utilize citrate in unique ways relative to mouse sperm.

In summary, these data suggest ejaculation is accompanied by an increase in metabolic flexibility. To fuel the Krebs cycle, epididymal mouse sperm are dependent upon pyruvate generated intrinsically from glycolysis, while uterine mouse sperm and ejaculated human sperm are able to fuel the Krebs cycle with exogenously supplied pyruvate (and citrate for human sperm). In all sperm samples capacitation is accompanied by an increase in oxphos activity. In sperm supplied with glycolytic substrates, this increase may reflect more glycolytically derived pyruvate fueling the Krebs cycle. However, in post-ejaculated sperm, pyruvate alone can support the capacitation-induced stimulation of oxphos implying that the rate of oxphos is independently stimulated during capacitation.

### Ejaculation provides higher flexibility in the carbon sources utilized for capacitation

**FIGURE 8:**
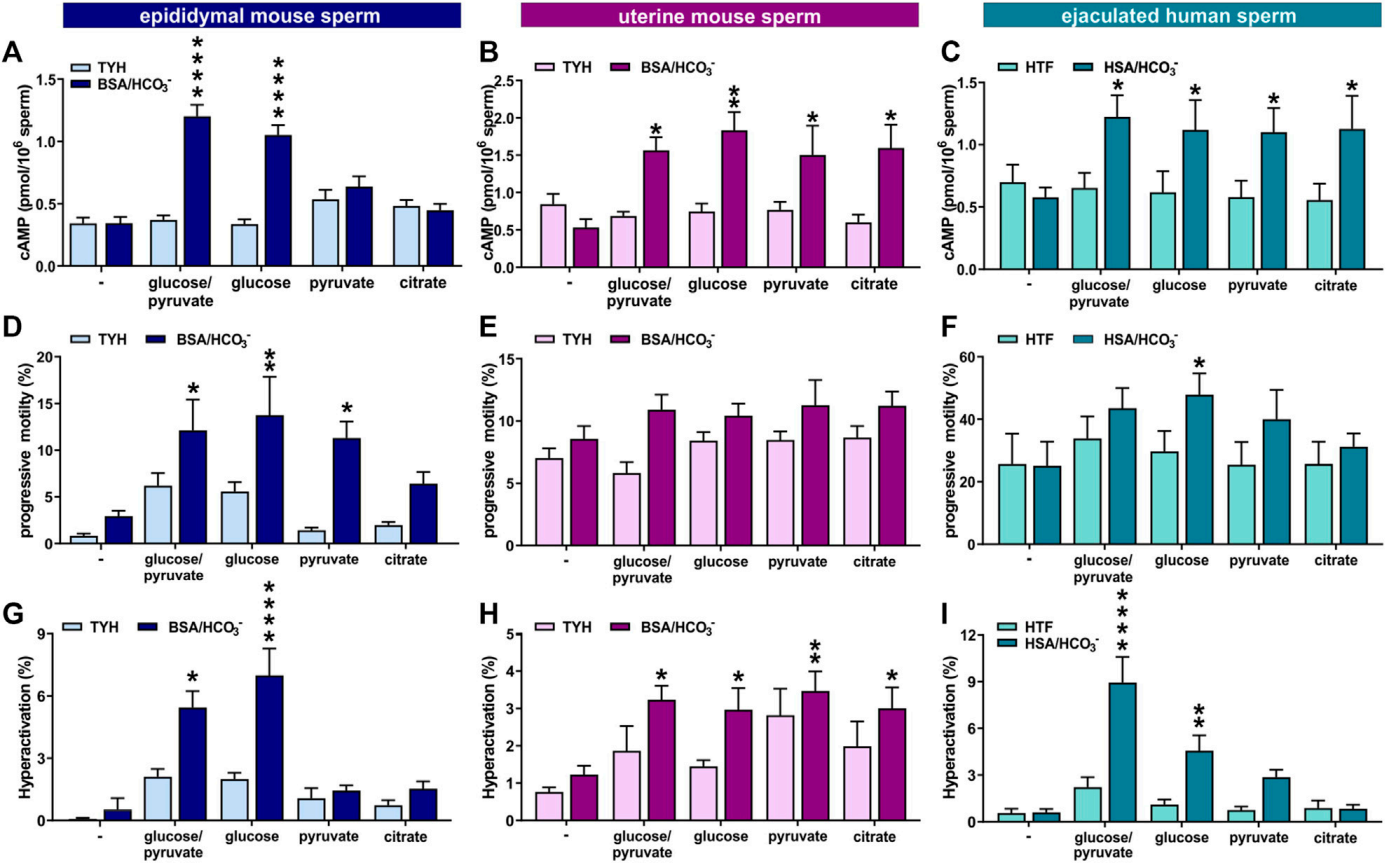
Ejaculation provides higher flexibility in the energy requirements for capacitation **(A–C)** Intracellular cAMP levels in **(A)** epididymal mouse sperm, **(B)** uterine mouse sperm, or **(C)** ejaculated human sperm incubated for 10 min (epididymal mouse sperm), 15 min (uterine mouse sperm), or 30 min (human sperm) in non-capacitating or capacitating media in the presence of no energy source (−), glucose/pyruvate, glucose, pyruvate, or citrate quantified by cAMP Elisa; mean +SEM (n ≥ 8) **(D–F)** Percentage of progressively motile **(D)** epididymal mouse sperm, **(E)** uterine mouse sperm, or **(F)** ejaculated human sperm incubated for 30 min (mouse) or 60 min (human) in non-capacitating or capacitating media in the presence of no energy source (−), glucose/pyruvate, glucose, pyruvate, or citrate; mean +SEM (n ≥ 7) **(G–I)** Percentage of hyperactivated **(G)** epididymal mouse sperm, **(H)** uterine mouse sperm, or **(I)** ejaculated human sperm incubated for 30 min (mouse) or 60 min (human) in non-capacitating or capacitating media in the presence of no energy source (−), glucose/pyruvate, glucose, pyruvate, or citrate; mean +SEM (n ≥ 7). Differences between conditions were analyzed using one-way ANOVA compared to the non-capacitated control in glucose,*p < 0.05, **p < 0.01, ****p < 0.0001.

Epididymal mouse sperm require glycolytic substrates to successfully undergo capacitation (Goodson et al., 2012; Balbach et al., 2020a), which may be at least partially due to their dependence on glycolytic substrates to support enhanced glycolysis and oxphos. Because uterine mouse sperm and ejaculated human sperm exhibit increased metabolic flexibility, we tested whether they are able to complete the molecular and physiological changes associated with capacitation in non-glycolytic substrates. The first event in the signaling cascade of capacitation is the bicarbonate-induced increase in cAMP. We measured capacitation-induced changes in intracellular cAMP in media with different energy sources at the time point with the maximal response (Balbach et al., 2021). As shown previously, epididymal mouse sperm only evoked an increase in intracellular cAMP in capacitating media when glucose was present. In contrast, and consistent with their enhanced metabolic flexibility, uterine mouse sperm and ejaculated human sperm increased cAMP production in both glycolytic and non-glycolytic substrates (Figures 8A–C).

The increased flexibility in carbon source utilization of uterine mouse and ejaculated human sperm also impacted motility. In contrast to epididymal mouse sperm under non-capacitating conditions, which seemed to require glucose for progressive motility (Figure 8D), uterine mouse and ejaculated human sperm incubated in non-capacitating conditions displayed equivalent levels of progressively motile sperm in glycolytic and non-glycolytic energy substrates. Interestingly, uterine mouse and ejaculated human sperm were progressively motile even in the absence of any exogenous energy source (Figures 8E, F). When incubated in capacitation-inducing conditions, all sperm samples again required an exogenous nutrient to support the increased energy demands of progressive motility and hyperactivation (Figures 8D–I). Epididymal mouse sperm showed similar increases in progressive motility induced by capacitating conditions with either glucose alone, or glucose in the presence of pyruvate, and a partial increase in the presence of citrate (Figure 8D and as shown in (Goodson et al., 2012)), but the switch to hyperactivated motility induced by capacitation-inducing conditions was only observed in the presence of glucose (Figure 8G). In contrast, each of the tested exogenous nutrients, glucose, glucose/pyruvate, pyruvate, or citrate, were able to support high levels of progressive motility and the capacitation-induced increase in hyperactivation in uterine mouse sperm. Thus, it appears the enhanced metabolic flexibility of ejaculated mouse sperm is functionally relevant; it afforded them the ability to leverage non-glycolytic energy substrates for energy consuming pathways. For ejaculated human sperm the response was more nuanced; the capacitation-induced increase in hyperactivated motility was absent in citrate, and while the response was present in pyruvate, glucose was superior with the strongest increase seen when combining glucose with pyruvate (Figures 8F, I), as observed previously (Hereng et al., 2011).

## Discussion

In mammals, the site of semen deposition is far from the site of fertilization, which means it is crucial for sperm to produce sufficient ATP to support their journey through the female genital tract. Mammalian sperm possess the molecular machinery to produce ATP via glycolysis and oxphos (Goldberg et al., 1977; Hess et al., 1993; Westhoff and Kamp, 1997; Narisawa et al., 2002; Miki et al., 2004; Nakamura et al., 2008; Danshina et al., 2010), and we now show that the activities of both pathways are stimulated during capacitation to meet the energy demands to successfully navigate the female reproductive tract. We find that human and mouse sperm depend upon both glycolysis and oxphos during capacitation to generate the additional energy required for progressive motility, which is only fully blocked when both pathways are inhibited. Additionally, our results indicate that for progressive and maximum hyperactivated motility, glycolysis and oxphos need to be simultaneously active.

Interestingly, ejaculated human sperm and post-ejaculated mouse sperm recovered from the uterus have the ability to leverage a variety of exogenous nutrients to fuel ATP generation which is different from mouse sperm isolated from the epididymis. Metabolic flexibility has been discussed previously as potentially beneficial for reproductive success (Travis et al., 2001; Miki, 2007; Storey, 2008; Tourmente et al., 2015; Tourmente et al., 2022a; Tourmente et al., 2022b). These data now suggest increased flexibility in carbon source utilization as a previously unappreciated change associated with sperm ejaculation.

Previous studies exploring the metabolism of mouse sperm started from dormant sperm isolated from the cauda epididymis, which were subsequently capacitated *in vitro*. In contrast, studies of human sperm used ejaculated sperm. Upon ejaculation, sperm are exposed to various energy substrates (Owen and Katz, 2005) and elevated levels of bicarbonate, which lead to changes in the sperm’s metabolic environment and initiate capacitation. Therefore, comparisons between mouse and human sperm metabolism were confounded by having (at least) two distinctions; species differences as well as the changes that accompany ejaculation. Here, we attempted to disambiguate these differences by comparing a third population of sperm; ejaculated mouse sperm isolated from the female reproductive tract. Because they are already exposed to the environment of the female reproductive tract, using mouse sperm isolated from the uterus introduces a new variable. However, uterine sperm represent a readily accessible source of sufficiently abundant ejaculated mouse sperm for the metabolic profiling studies presented here.

We subjected these three sperm samples to two complementary methods for characterizing metabolism, extracellular flux analysis, and metabolic profiling. Extracellular flux analysis provides information about real-time changes in glycolysis and oxphos; these experiments inform how rates of these metabolic pathways change during the process of capacitation. Targeted metabolic profiling measures the levels of specific endogenous metabolites which allows comparison of their relative levels before and after incubation in capacitating conditions. As seen with cAMP, which increased during capacitation, and ATP, which decreased during capacitation, changes in metabolite levels in either direction can reflect capacitation-associated changes in the enzymatic activities producing and/or consuming metabolites.

When using these methods to compare ejaculated human sperm to uterine mouse sperm, we observed more similarities than differences between the two species. Measuring changes in glycolysis and oxphos in real-time revealed that the rate of both glycolysis and oxphos increase during mouse and human sperm capacitation. An increase in mitochondrial activity during capacitation of epididymal mouse sperm was independently observed using flow cytometry (Giaccagli et al., 2021) and high-resolution respirometry (Ferreira et al., 2021b). In contrast, a study by Tourmente *et al.* observed a shift in the usage ratio from oxphos to glycolysis (Tourmente et al., 2022a) instead of an increase in the rate of oxphos and glycolysis. In their study, to overcome the limitation that bicarbonate cannot be included during incubations in the extracellular flux analyzer, Tourmente *et al.* pre-incubated epididymal mouse sperm in capacitating conditions in advance of the experiment and measured extracellular flux from sperm incubated in non-capacitating media. Since sperm capacitation is reversible (Cohen-Dayag et al., 1995; Navarrete et al., 2019), the absence of bicarbonate during the actual measurement makes the sperm maturation state unclear. Moreover, instead of monitoring physiologically induced changes in glycolysis and oxphos over time, in their study the authors extrapolate the contribution of glycolysis and oxphos from addition of pharmacological inhibitors of the two pathways. These differences between the experimental design of both studies make a direct comparison difficult.

The enhanced metabolic flexibility seen in sperm post ejaculation was observed under both non-capacitating as well as capacitating conditions. In contrast to epididymal mouse sperm, which required glycolytic substrates to support progressive motility, progressive motility in uterine mouse sperm under non-capacitating conditions was independent of exogenous nutrients. This suggests that uterine mouse sperm are able to leverage stored energy or secure sufficient nutrients from an initial exposure to energy substrates during ejaculation or in the female genital tract. In capacitating conditions, the bicarbonate-induced increase in progressive motility and hyperactivation demanded exogenous energy sources, and uterine mouse sperm, unlike epididymal sperm, were able to support hyperactivation using the non-glycolytic exogenous substrates pyruvate and citrate. Consistently, metabolic flux analysis revealed that uterine sperm can use pyruvate to support the capacitation-associated increase in the rate of oxphos. Because we observed similar tendencies in ejaculated human sperm, we posit that ejaculation equips sperm with enhanced metabolic flexibility. Importantly, this also suggests that the rate of oxphos is not just passively adapting to an increase in substrate availability generated by enhanced glycolytic activity. Instead, ejaculated sperm seem to actively increase the flux through oxphos during capacitation. Since sperm are transcriptionally silent and exist in a constantly changing environment with fluctuating O_2_ tensions and energy substrate availability, bioenergetic adaptability would be beneficial for enabling sperm to achieve their ultimate function: fertilization of the oocyte.

## Methods

### Reagents

Reagents for media preparation were purchased from Sigma-Aldrich. 2-deoxyglucose (2-DG), 3-Isobutyl-1-methylxanthine (IBMX), antimycin A, BSA, concanavalin A (ConA), dibutyryl-cAMP (db-cAMP), progesterone, lectin from Pisum sativum FITC-conjugated (PSA-FITC) and lectin from *Arachis hypogaea* FITC-conjugated (PNA-FITC) were purchased from Sigma-Aldrich, rotenone (rot) from Cayman chemicals, HSA from Irvine Scientific and PBS buffer from Corning.

### Mice

Adult 8–10 week old C57BL/6N (Stock #: 027) mice were purchased from Jackson Laboratory and allowed to acclimatize before use. Animal experiments were approved by Weill Cornell Medicine’s and by the University of Massachusetts Institutional Animal Care and Use Committees (IACUC).

### Sperm preparation

Epididymal mouse sperm were isolated by incision of the cauda epididymis in 500 µL modified TYH medium (in mM: 135 NaCl, 4.7 KCl, 1.7 CaCl_2_, 1.2 KH_2_PO_4_, 1.2 MgSO_4_, 10 HEPES, pH 7.4 adjusted at 37°C with NaOH) without energy source, prewarmed at 37°C. For Seahorse experiments, Seahorse TYH buffer with 1 mM HEPES was used for isolation. For assessment of ejaculated mouse sperm, single-housed male and female mice were acclimatized to a reverse light cycle (dark: 11 a.m. to 11 p.m.) for at least 2 weeks. On the day of the experiment at 11 a.m. individual males were paired with a female in estrus (identified by physical examination) and allowed to mate until a plug was discovered. Female mice were sacrificed, the uteri were isolated and both uterine horns opened in 1 mL TYH buffer without energy source. After 15 min swim-out at 37°C, sperm were counted using a hematocytometer and sperm cell numbers were adjusted to a concentration of 1 × 10^7^ cells/mL. Sperm were washed twice with the TYH buffer with the respective energy source used for the experiment (glucose (5.6 mM), glucose (5.6 mM) and pyruvate (0.56 mM), pyruvate (0.56 mM), or citrate (0.56 mM)) by centrifugation at 700 x g for 5 min. For capacitation, sperm were incubated in TYH containing 3 mg/mL BSA and 25 mM NaHCO_3_ in a 37°C incubator. For seahorse experiments, NaHCO_3_ was replaced with 5 mM db-cAMP and 500 µM IBMX.

Samples of human semen were obtained from healthy volunteers with their prior written consent following a protocol approved by Weill Cornell Medicine’s Institutional Review Board (IRB 21-03023495). Only samples that met the WHO 2010 criteria for normal semen parameters (ejaculated volume ≥1.5 mL, sperm concentration ≥15 million/mL, motility ≥40%, progressive motility ≥32%, normal morphology ≥4%) were included in this study. Semen was incubated for 30 min in a 37°C incubator to liquefy. Human sperm were purified by “swim-up” procedure in human tubular fluid (HTF) (in mM: 97.8 NaCl, 4.69 KCl, 0.2 MgSO_4_, 0.37 KH_2_PO_4_, 2.04 CaCl_2_, 20 HEPES, pH 7.4) with the respective energy source (glucose (2.8 mM), glucose (2.8 mM) and pyruvate (0.33 mM), pyruvate (0.33 mM), or citrate (0.33 mM)) used for the experiment. For Seahorse experiments, Seahorse HTF buffer with 1 mM HEPES was used. 0.5–1 mL of liquefied semen was layered in a 50 mL tube below 4 mL HTF. The tubes were incubated at a tilted angle of 45° at 37°C for 60 min. Motile sperm were allowed to swim up into the HTF layer; immotile sperm and other cells or tissue debris remain in the ejaculate fraction. Up to 3 mL of the HTF layer was transferred to a fresh tube and washed twice by centrifugation (700 × g, 20 min) with HTF buffer with the respective energy source. The purity and vitality of each sample was assessed via light microscopy. Sperm cell numbers were determined using a hemocytometer and adjusted to a concentration of 1 × 10^7^ cells/mL. For capacitation, sperm were incubated in HTF with 72.8 mM NaCl containing 25 mM NaHCO_3_ and 3 mg/mL human serum albumin (HSA) in a 37°C incubator. For seahorse experiments, NaHCO_3_ was replaced with 5 mM db-cAMP and 500 µM IBMX.

### Sperm motility assays

Sperm motility as assessed using computer-assisted sperm analysis (CASA) via Hamilton–Thorne digital image analyzer (IVOS II, Hamilton Thorne Research, Beverly, MA) with the following parameters: 30 frames, frame rate: 60 Hz, cell size: 30–170 μm^2^. Sperm movements of 5 fields of at least 200 sperm were examined. For mouse sperm analysis, following incubation for 30 min in non-capacitating or capacitating TYH buffer, 25 µL sperm suspension were loaded on a 100 μM Leja slide (Hamilton Thorne) and placed on a microscope stage at 37 °C. Mouse sperm were considered progressively motile when presenting STR ≥80% and VAP ≥50 μm/s and hyperactivated when presenting VCL ≥270 μm/s, LIN <50%, and ALH ≥7 μm. For human sperm analysis, following incubation for 60 min in non-capacitating or capacitating HTF buffer, 8 µL sperm suspension were loaded on a 20 μM Leja slide. Human sperm were considered progressively motile when presenting STR ≥80% and VAP ≥25 μm/s and hyperactivated when presenting VCL ≥150 μm/s, LIN <50%, and ALH ≥7 μm. Data is shown as percentage of the total cell number.

### Acrosome reaction assay

For analysis of acrosomal exocytosis, 100 µL of 1 × 10^6^ sperm/mL were capacitated for 90 min in TYH buffer supplemented with 3 mg/mL BSA and 25 mM NaHCO_3_ or 3 mg/mL BSA, 5 mM db-cAMP and 500 μM IBMX (mouse sperm) or HTF buffer supplemented with 3 μL/mL HSA and 25 mM NaHCO_3_ or 3 μL/mL HSA, 5 mM db-cAMP and 500 μM IBMX (human sperm). Acrosome reaction was induced by incubation with 10 μM progesterone for 15 min at 37 °C. The sperm suspensions were sedimented by centrifugation at 2,000xg for 5 min and the sedimented sperm were resuspended in 100 µL PBS buffer. Samples were air-dried on microscope slides and fixed for 30 min in 100% ethanol at room temperature. For acrosome staining, mouse and human sperm were incubated for 30 min in the dark with 5 μg/mL PNA-FITC or 5 μg/mL PSA-FITC, respectively, and counterstained with 2 μg/mL DAPI. After curing, slides were analyzed using a Zeiss LSM 880 Laser Scanning Confocal Microscope; images were captured with two photomultiplier and one Gallium Arsenide Phosphide detector using ZEN Imaging software. For each condition, at least 600 cells were analyzed using ImageJ 1.52.

### Extracellular flux analysis

Extracellular flux analysis was performed as described in (Balbach et al., 2020a; Balbach et al., 2020b). In brief, 180 µL mouse and human sperm suspensions in Seahorse TYH/HTF buffer with 3 mg/mL BSA or HSA and the respective energy source were added to each well of a ConA-coated 96-well plate (8.3 × 10^5^ sperm/well). To immobilize the sperm heads to the well bottom, the plate was centrifuged at 250 × g for 1 min, rotated by 180°, and centrifuged again. For experiments comparing different energy sources, after recording a baseline and a buffer injection with the respective buffer, db-cAMP/IBMX was injected into half of the wells to induce capacitation and changes in ECAR and OCR were monitored for 90 min. Alternatively, after the buffer injection, first 2-deoxyglucose (2-DG) or AntimycinA (AntA)/Rotenone (Rot) were injected followed, after 3 measurement cycles, by db-cAMP/IBMX to induce capacitation.

Data was analyzed as described in (Goodson et al., 2012) and is shown as fold change normalized to the data point before cAMP/IBMX injection. The fold change shown in the bar graphs was generated by normalizing the mean of the last three data points in capacitating conditions with the mean of the last three data points of non-capacitated sperm.

### ATP quantification

Aliquots of 1 × 10^6^ sperm were incubated for the indicated time points in non-capacitating or capacitating TYH buffer with glucose/pyruvate (mouse sperm) or non-capacitating or capacitating HTF buffer with glucose/pyruvate (human sperm) in the absence or presence of 2-DG. Sperm were sedimented by centrifugation at 2000 × g for 3 min and lysed in 250 µL lysis buffer (100 mM Tris, 4 mM EDTA, pH 7.75) at 95°C for 7 min. Sperm lysates were centrifuged at 10000xg for 3 min and the ATP in the supernatant was quantified using the ENLITEN ATP Assay Kit (Promega) according to the manufacturer’s instructions.

### Sample preparation for LC-MS/MS

Epididymal and uterine mouse sperm and ejaculated human sperm (5 × 10^6^ sperm/mL) were isolated and incubated in non-capacitating and capacitating conditions for 90 min in quadruplicates in 500 μL TYH or HTF media with glucose or glucose/pyruvate. Following separation of the supernatant and sperm by centrifugation for 5 min at 700 × g, 450 μL of supernatant were transferred to a new vial and 450 μL ice-cold 90% MeOH were added to the sperm pellet resulting in a final concentration of 80% methanol (MeOH). For the supernatant samples, 100 μL were quenched with 400 μL 100% MeOH. Media without sperm was used as control and processed under the same conditions. After the addition of MeOH, samples were immediately vortexed and placed on dry ice. After 15 min, samples were moved to wet ice, vortexed again and incubated overnight at −80°C to aid protein precipitation. Sperm and supernatant samples were centrifuged at 20,000 × g for 20 min at 4°C and 450 μL of supernatant were transferred to a fresh tube, dried via speed-vac and stored at −80°C until analysis. Before LC-MS/MS analysis, the samples were resuspended in 30 μL of mobile phase A and incubated on wet ice for 20 min while being vortexed every 5 min. Samples were centrifuged at 20,000 × g for 20 min at 4°C and 25 μL of the supernatant was transferred into LC vials for injection.

### LC-MS/MS analysis

Ion pair LC-MS/MS analysis was performed with LC separation on a Zorbax RRHD Extend-C18 column (150 mm × 2.1 mm, 1.8 μm particle size, Agilent Technologies), and using gradient of solvent A (10 mM tributylamine and 15 mM acetic acid in 97:3 water:methanol) and solvent B (10 mM tributylamine and 15 mM acetic acid in methanol) according to the manufacturer’s instructions (MassHunter Metabolomics dMRM Database and Method, Agilent Technologies).

### cAMP quantification

Aliquots of 2 × 10^6^ epididymal mouse sperm were incubated for 10 min in non-capacitating or capacitating TYH buffer with the respective energy source, aliquots of 2 × 10^6^ uterine mouse or ejaculated human sperm were incubated for 15 or 30 min in non-capacitating or capacitating TYH or HTF buffer, respectively. Sperm were sedimented by centrifugation at 2000 × g for 3 min and lysed in 200 µL HCl for 10 min. Sperm lysates were centrifuged at 2000 × g for 3 min and the cAMP in the supernatant acetylated and quantified using the Direct cAMP ELISA Kit (Enzo) according to the manufacturer’s instructions.

### Statistical analysis

Statistical analyses were performed using GraphPad Prism 5 (Graph-Pad Software). All data are shown as the mean ± SEM. Statistical significance between two groups was determined using two-tailed, paired t-tests with Welch’s correction, statistical significance between multiple groups was determined using one-way ANOVA with Dunnett’s correction after confirming normal distribution via D'Agostino-Pearson and homoscedasticity (Breusch–Pagan test). Differences were considered to be significant if ^∗^ *p* < 0.05, ^∗∗^ *p* < 0.01, ^∗∗∗^ *p* < 0.001, and ^∗∗∗∗^ *p* < 0.0001.

## Data Availability

The original contributions presented in the study are included in the article/Supplementary Material, further inquiries can be directed to the corresponding author.
