# Supplementary material for: Capacitation induces changes in metabolic pathways supporting motility of epididymal and ejaculated sperm
Source: Front Cell Dev Biol. 2023 Jun 27;11:1160154. doi: 10.3389/fcell.2023.1160154 (PMC10335746; doi:10.3389/fcell.2023.1160154)
Supplement: Supplementary file 4 [file DataSheet1.PDF]

Supplementary Material

Capacitation induces changes in metabolic pathways supporting motility of epididymal and ejaculated sperm

Melanie Balbach[1]*, Lubna Ghanem[1], Sara Violante[2], Aye Kyaw[2], Ana Romarowski[3], Justin R Cross[2], Pablo E Visconti[3], Lonny R. Levin[1] & Jochen Buck[1]

[1]Department of Pharmacology, Weill Cornell Medical College, New York City, NY

[2]Donald B. and Catherine C. Marron Cancer Metabolism Center, Memorial Sloan Kettering Cancer Center, New York City, NY

[3]Department of Veterinary and Animal Science, Integrated Sciences Building, University of Massachusetts, Amherst, MA

To whom correspondence should be addressed:

Dr. Melanie Balbach, Department of Pharmacology, Weill Cornell Medical College, 1300 York Avenue, 10044 New York City, NY; phone: +1 212 746 6274, email: meb2023@med.cornell.edu

Supplementary Figure S1

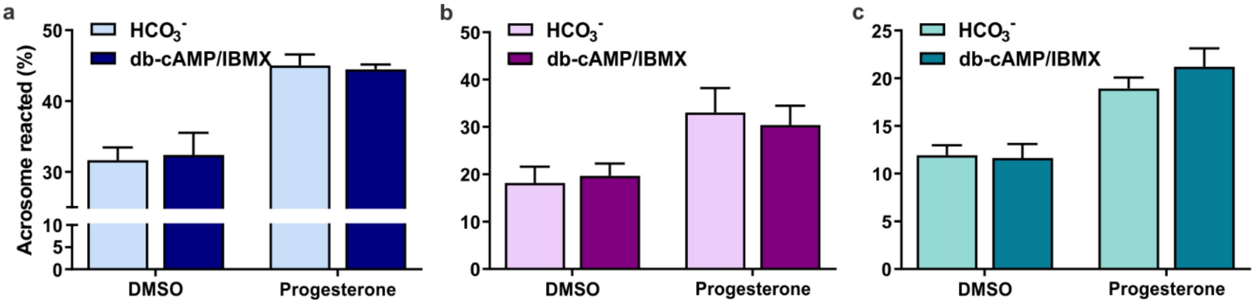

Fig. S1: Progesterone-induced acrosome reaction is comparable in traditional capacitation and Seahorse capacitation media

(a-c) Acrosome reaction evoked by 10 µM progesterone in **(a)** epididymal mouse sperm, **(b)** uterine mouse sperm, or **(c)** ejaculated human sperm incubated for 90 min in capacitating media with 25 mM HCO_3^- and 3 mg/ml BSA (mouse) or HSA (human) or Seahorse capacitating media with 5 mM db-cAMP, 500 µM IBMX and 3 mg/ml BSA; mean + SEM (n=5). Differences between sperm capacitated in HCO_3^- or db-cAMP/IBMX were analyzed using two-tailed, paired *t*-test, p=not significant.

Supplementary Figure S2

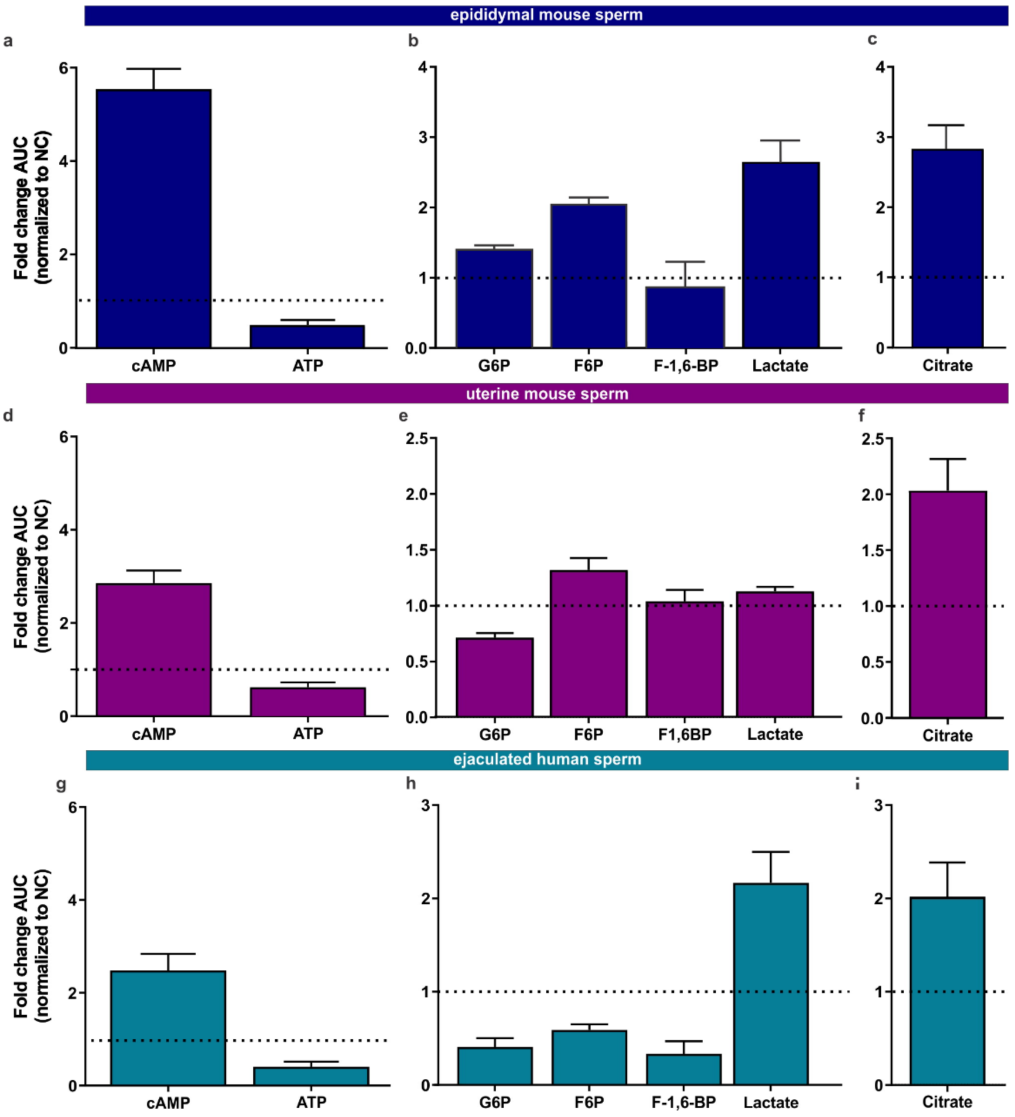

Fig. S2: Metabolic profiling of mammalian sperm in non-capacitated and capacitating conditions in glucose and pyruvate

(a,d,g) Nucleotides detected in **(a)** epididymal mouse sperm, **(d)** uterine mouse sperm, or **(g)** ejaculated human sperm incubated for 90 min in capacitating conditions in glucose/pyruvate, fold change AUC normalized to the non-capacitated control; mean + SEM (n=3). **(b,e,h)** Glycolytic metabolites detected in **(b)** epididymal mouse sperm, **(e)** uterine mouse sperm, or **(h)** ejaculated human sperm incubated for 90 min in capacitating conditions in glucose/pyruvate, fold change AUC normalized to the non-capacitated control; mean + SEM (n=3). **(c,f,i)** Citrate detected in **(c)** epididymal mouse sperm, **(f)** uterine mouse sperm, or **(i)** ejaculated human sperm incubated for 90 min in capacitating conditions in glucose/pyruvate, fold change AUC normalized to the non-

capacitated control; mean + SEM (n=3). G6P = glycerol-6-phosphate, F6P = fructose-6-phosphate, F-1,6-BP = fructose 1,6-bisphosphate.

Supplementary Figure S3

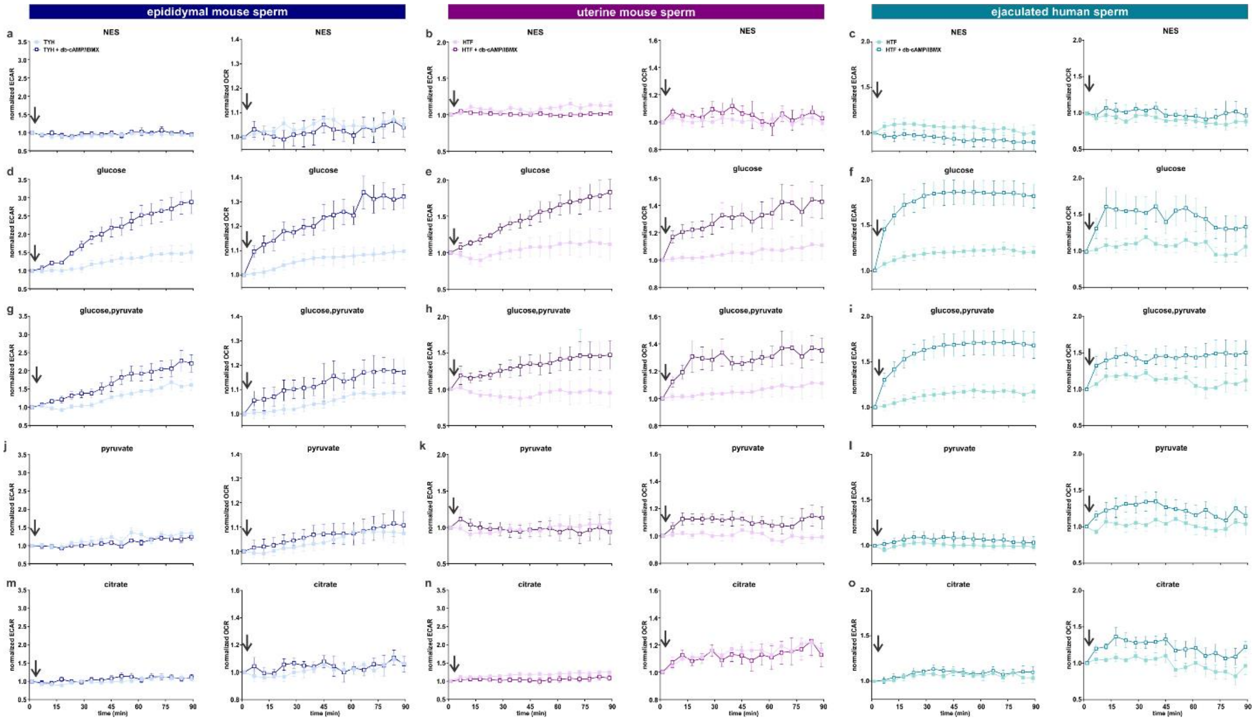

Fig. S3: Capacitation-induced changes in metabolism in glycolytic substrates

(a,b,c) Normalized ECAR (left) and OCR (right) of **(a)** epididymal mouse sperm, **(b)** uterine mouse sperm, or **(c)** ejaculated human sperm in non-capacitating or capacitation-inducing media without energy source (NES); mean ± SEM (n≥7). **(d,e,f)** Normalized ECAR (left) and OCR (right) of **(d)** epididymal mouse sperm, **(e)** uterine mouse sperm, or **(f)** ejaculated human sperm in non-capacitating or capacitation-inducing media with glucose; mean ± SEM (n≥7). **(g,h,i)** Normalized ECAR (left) and OCR (right) of **(g)** epididymal mouse sperm, **(h)** uterine mouse sperm, or **(i)** ejaculated human sperm in non-capacitating or capacitating media with glucose/pyruvate; mean ± SEM (n≥7). **(j,k,l)** Normalized ECAR (left) and OCR (right) of **(j)** epididymal mouse sperm, **(k)** uterine mouse sperm, or **(l)** ejaculated human sperm in non-capacitating or capacitation-inducing media with pyruvate; mean ± SEM (n≥7). **(m,n,o)** Normalized ECAR (left) and OCR (right) of **(m)** epididymal mouse sperm, **(n)** uterine mouse sperm, or **(o)** ejaculated human sperm in non-capacitating or capacitation-inducing media with citrate; mean ± SEM (n≥7). Arrow indicates addition of 5 mM db-cAMP/500 µM IBMX.

Supplementary Table S1: Basal ECAR and OCR of non-capacitated epididymal and uterine mouse sperm and ejaculated human sperm in media with different energy substrates (n≥13, mean ± SEM)

	Epididymal mouse sperm	Uterine mouse sperm	Ejaculated human sperm
**ECAR glucose (mpH/min)**	12.7 ± 1.6	25.6 ± 1.7	21 ± 1.6
**OCR glucose (pmol/min)**	33.1 ± 5.9	51.4 ± 3.6	52.8 ± 2.5
**ECAR glucose/pyruvate (mpH/min)**	6.8 ± 1.5	16.5 ± 1.8	11.9 ± 1.9
**OCR glucose/pyruvate (pmol/min)**	16.4 ± 1.4	32.2 ± 2.3	35.4 ± 4.5
**ECAR pyruvate (mpH/min)**	11.1 ± 2.2	17.4 ± 2.5	9.4 ± 1.1
**OCR pyruvate (pmol/min)**	15.1 ± 4.5	34.4 ± 5.7	32.6 ± 2.6
**ECAR citrate (mpH/min)**	4.4 ± 1.3	9.8 ± 1.3	10.8 ± 1.9
**OCR citrate (pmol/min)**	13.3 ± 1.6	16.0 ± 3.3	32.2 ± 2.3
**ECAR no energy source (mpH/min)**	2.6 ± 0.6	4.7 ± 1.9	7.4 ± 1.3
**OCR no energy source (pmol/min)**	11.7 ± 1.7	13.6 ± 2.7	17.2 ± 5.7
